# Structural modulation of silicon nanowires by combining a high gas flow rate with metal catalysts

**DOI:** 10.1186/s11671-015-0893-4

**Published:** 2015-04-21

**Authors:** Dongjea Seo, Jaejun Lee, Sung Wook Kim, Ilsoo Kim, Jukwan Na, Min-Ho Hong, Heon-Jin Choi

**Affiliations:** Department of Materials Science and Engineering, Yonsei University, Seoul, 120-749 Korea

**Keywords:** Structural modulation, SiNW, Vapor-liquid-solid, Growth, Catalyst

## Abstract

We grew silicon nanowires (SiNWs) by a vapor-liquid-solid (VLS) mechanism using metal catalysts of gold (Au), titanium (Ti), manganese (Mn), and iron (Fe) under a high flow rate of hydrogen (H_2_). This combination of catalyst types and high gas flow rate revealed the potential for growing various SiNWs, including kinked SiNWs (with Au), ultra-thin SiNWs having diameters about 5 nm (with Ti), rough-surfaced SiNWs (with Mn), and ribbon-shaped SiNWs tens of microns in width (with Fe). The high flow rate of gas affects the VLS mechanism differently for each combination; for example, it induces an unstable solid-liquid interfaces (with Au), active etching of the catalyst (with Ti), sidewall deposition by a vapor-solid (VS) mechanism, and an asymmetric precipitation of Si in the catalyst (with Fe). Our combinatorial approach may provide a new path for the structural modulation of SiNWs via the VLS mechanism.

**PACS:** 80; 81; 82

## Background

Silicon nanowires (SiNWs) have considerable potential for several applications including electronics [1-4], sensors [5-7], energy conversion [8-11], and photonic devices [12,13] owing to their novel physical and chemical properties. Meanwhile, the structural modulation of NWs is critical to exploit their potential and realize devices with high performance [14-16]. For example, kinked [17-20] and branched [21,22] SiNWs have advantages in some applications due to their higher surface-to-volume ratio. SiNWs are generally grown by the vapor-liquid-solid (VLS) mechanism with the assistance of metal catalysts [23-25]. This mechanism is simple and versatile for SiNW preparation; however, structural modulation using this method is difficult because the liquid catalysts have a globular shape resulting from surface tension, which confines the shape of the NWs to a rod form, limiting further shape or size variation. Some structural modification of SiNWs during their growth has been studied by changing the pressure or temperature [26]; however, these alterations have shown only limited success, for example, the growth of kinked nanowires [27-29]. And catalyst diffusion during the growth determines the shape of nanowire [30].

Here, we report on the growth of various SiNWs using metal catalysts of gold (Au), titanium (Ti), manganese (Mn), and iron (Fe) under a high flow rate of hydrogen (H_2_). Through the combination of different types of catalyst and a high flow rate of gas, we could grow structurally modulated SiNWs that are kinked, ultra thin, with a rough surface, or have a thin ribbon shape. The results indicate that the high flow rate of H_2_ significantly affects the growth mode behavior, making room for the structural modulation of SiNWs via the VLS mechanism.

## Methods

The SiNWs were grown on Si (100) substrates. The substrates were degreased with solvent, etched in a HF solution, and rinsed with deionized H_2_O. The substrates were then immersed in a diluted hydrofluoric acid solution (HF:H_2_O = 1:9). Two approaches were initially investigated for positioning the catalyst on the substrates to choose the ideal approach for the growth of NWs: (1) deposition of metal films and (2) supplying the metal component in the vapor phase by evaporation of metal precursors. In the case of the Au and Ti catalysts, metal layers approximately 5 nm (Au) and 2 nm (Ti) thick were deposited on the substrates. In the case of the Mn and Fe catalysts, each was supplied to the substrates through the gas phase using MnCl_2_ (99.99%, Sigma-Aldrich, St. Louis, MO, USA) or FeI_2_ (99.99%, Sigma-Aldrich, St. Louis, MO, USA) powders as a precursor for the metal catalyst. The SiNWs were grown in a hot wall quartz tube reactor under a H_2_ flow rate of 300 to 3,000 sccm∙min^−1^ using Si tetrachloride (SiCl_4_) (99.9999%, Sigma-Aldrich, St. Louis, MO, USA) as the Si source at 1,050°C for 30 min. The morphologies of the SiNWs were characterized by scanning electron microscopy (SEM) and high-resolution transmission electron microscopy (HRTEM).

## Results and discussion

**Figure 1:**
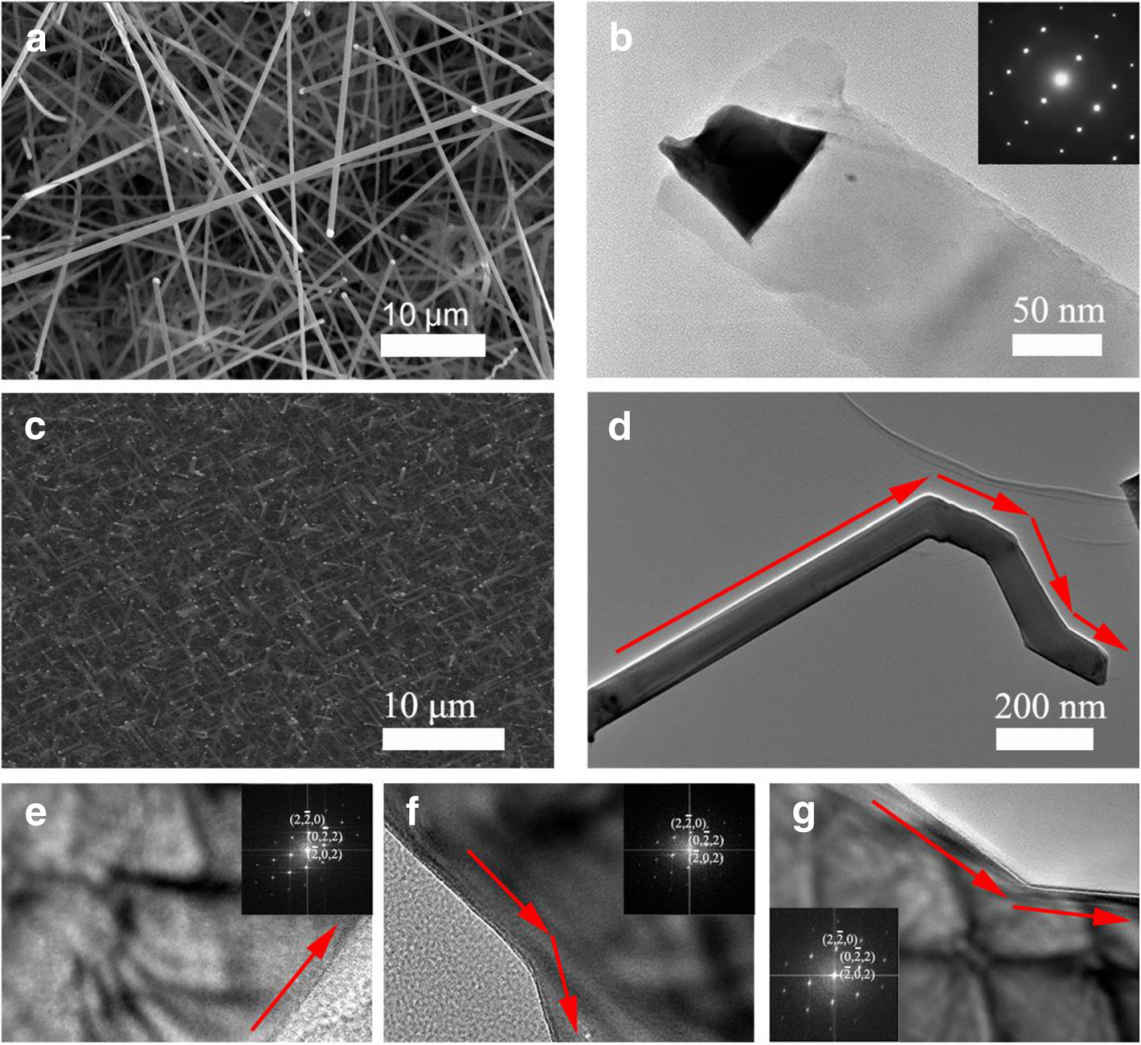
Synthesis of general SiNWs and kinked SiNWs. **(a)** SEM image of a general silicon nanowire grown on Si (100) substrates. The faceting along the side facets of SiNWs is clearly visible as well as the gold (Au) catalyst at **(b)**, TEM image of general SiNWs and inset showed SAED pattern images of SiNWS. **(c)** SEM image of a kinked SiNWS on Si (100) substrate with high flow rate of hydrogen (3,000 sccm∙min^−1^). **(d)** TEM image shows that kinked SiNWs were grown which kink away of the growth direction of NWs from [101] direction. **(e-g)**, HRTEM image of the same kinked SiNWs, showing initial growth direction [121], kinking away from <101 > to the other <101 > directions.

A general method to obtain SiNWs is based on VLS method with gold catalyst [31-33]. The gold is a metal that works well as catalyst compared to other metals. Figure 1a shows an SEM image of typical SiNWs grown on a Si substrate using an Au catalyst. Under a low gas flow rate, e.g., 300 sccm∙min^−1^, straight SiNWs with smooth surfaces were grown, as reported in numerous previous studies. Transmission electron microscopy (TEM) investigation revealed that the SiNW growth direction was [113]. A HRTEM image (Figure 1b) shows that the SiNWs are single crystalline. The globule at the tip of the NWs indicates the working of VLS mechanism for the growth of NWs. In this system, low gas flow rate and interface between Au-Si alloy and Si were stable during the growth. And SiNWs confined the shape of the NWs to a flat rod form due to a globular shape of the liquid Au catalyst.

Figure 1c,d shows the SiNWs grown under a high flow rate of H_2_ gas (3,000 sccm∙min^−1^). These show that primarily kinked SiNWs were grown on the substrate. Figure 1e,f,g shows that the SiNW growth direction is [101], and the kinks are away from the [101] to other <101 > directions. In previous studies, kinked SiNWs were grown by modulating the pressure, displaying a kinking between two [112] oriented segments at defined positions along the length of the NWs [34-36]. The d spacing measured from the Fourier transform of TEM is 3.103 Å for the (112) plane and 3.155 Å for the (110) plane, respectively, that are within 2% of the values of pure Si. The composition analysis using energy-dispersive X-ray analysis (EDAX) in TEM also showed only Si without any impurities.

As a result of the liquid-solid interface instability in the VLS mechanism, the amount of the Si component supplied to the liquid catalysts during the course of growth can be changed by differences in pressure. Similarly, the high flow rate of the H_2_ supplies less of the Si component to the liquid catalysts since such a high flow rate dilutes the precursor concentration in the gas phase. This induces instability of the liquid alloy-solid interface due to a dilute Si concentration in the catalysts, leading to an irregular stacking of Si at the interfaces, kinking the growth direction.

**Figure 2:**
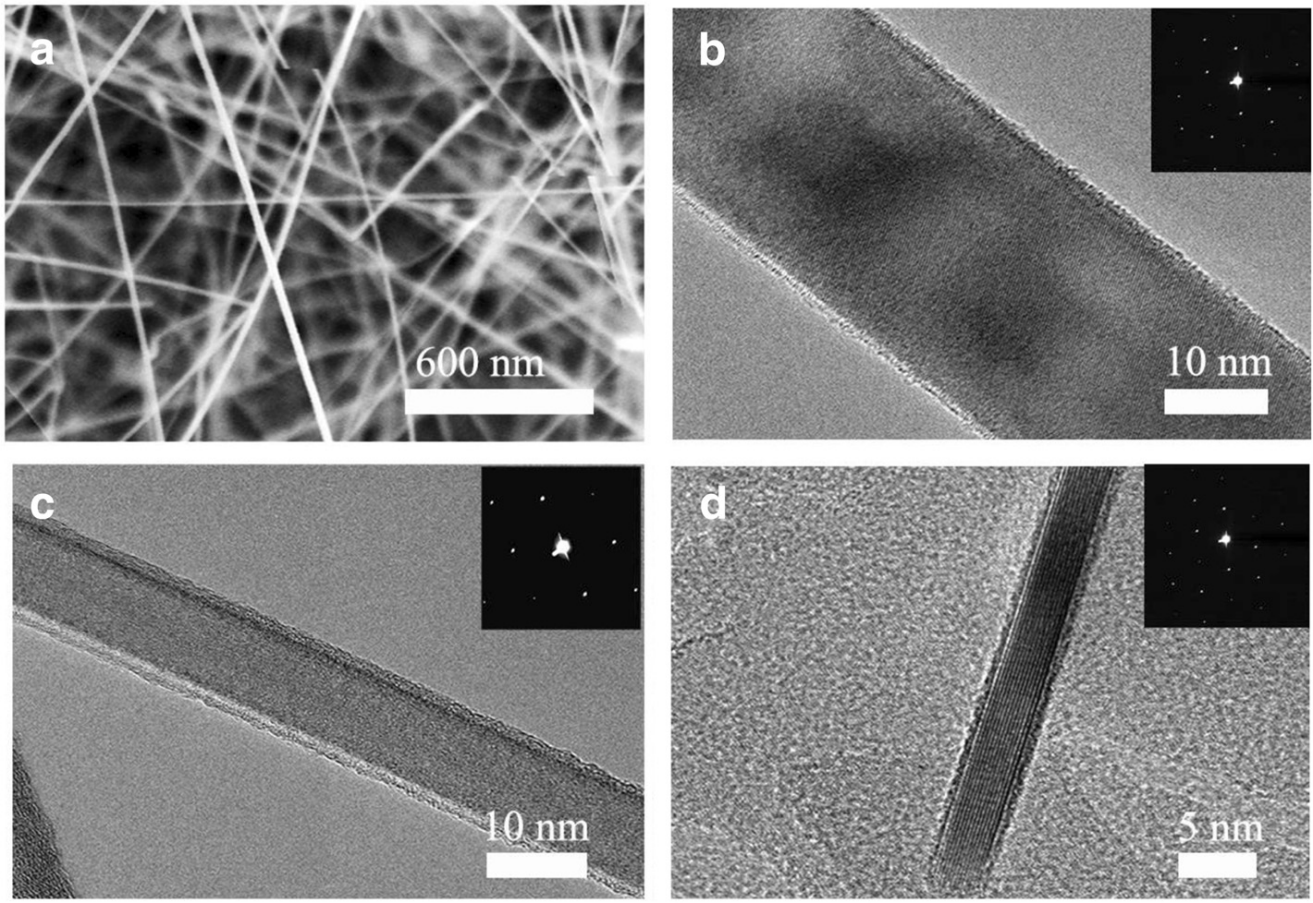
Thin SiNWS grown by using Ti as catalyst. **(a)** SEM image of thin SiNWs with high flow rate of gas (H_2,_ 3,000 sccm∙min^−1^). HRTEM images of thin SiNWs. It shows that all the thin SiNWs are single crystal and the growth direction is [113] **(b-d)**. That is the diameter becomes smaller with the flow rate of hydrogen. **(b)** 1,000 sccm∙min^−1^, **(c)** 2,000 sccm∙min^−1^, and **(d)** 3,000 sccm∙min^−1^.

Figure 2 shows the SiNWs grown using Ti as the catalyst. As shown, the diameter of SiNWs ranged from 5 to 30 nm. It should be noted that the diameter is less than those formed with Au catalysts. It should also be noted that the diameter correlates to the H_2_ gas flow rate, becoming smaller with an increased flow rate. As shown in Figure 2b,c,d, the SiNWs have diameters of about 30 ± 10 nm with a 1,000 sccm∙min^−1^ hydrogen flow rate, 20 ± 5 nm with 2,000 sccm∙min^−1^, and 5 ± 1 nm with 3,000 sccm∙min^−1^. The d spacing measured from the Fourier transform of TEM and the composition analysis using EDAX in TEM showed only Si without any impurities or silicides.

It was revealed that Ti catalyst is essential for the growth of SiNWs since any NWs were not grown on the substrate without Ti catalysts. However, liquid globules were not found at the tip of the SiNWs grown with Ti. This indicates that Ti worked as a catalyst at the early stage of the VLS mechanism before being etched during the H_2_ flow with SiCl_4_ growth by the HCl by product gas, disappearing in the NWs. We already reported that Pt-Si liquid globules were etched out under a chloride atmosphere [37]. It is similar tendency to using the Ti catalyst. The etching of Ti in chlorinated environments has been reported in the literature [38,39]. It can also be assumed that the etching rate is proportional to the gas flow rate. Therefore, the etching is significant with the high gas flow rate, leading to a smaller size of the Ti catalyst, which resulted in smaller SiNWs. It should be noted that very thin SiNWs with sub-10 nm diameters could be easily obtained by the combination of a high gas flow rate and Ti catalyst.

**Figure 3:**
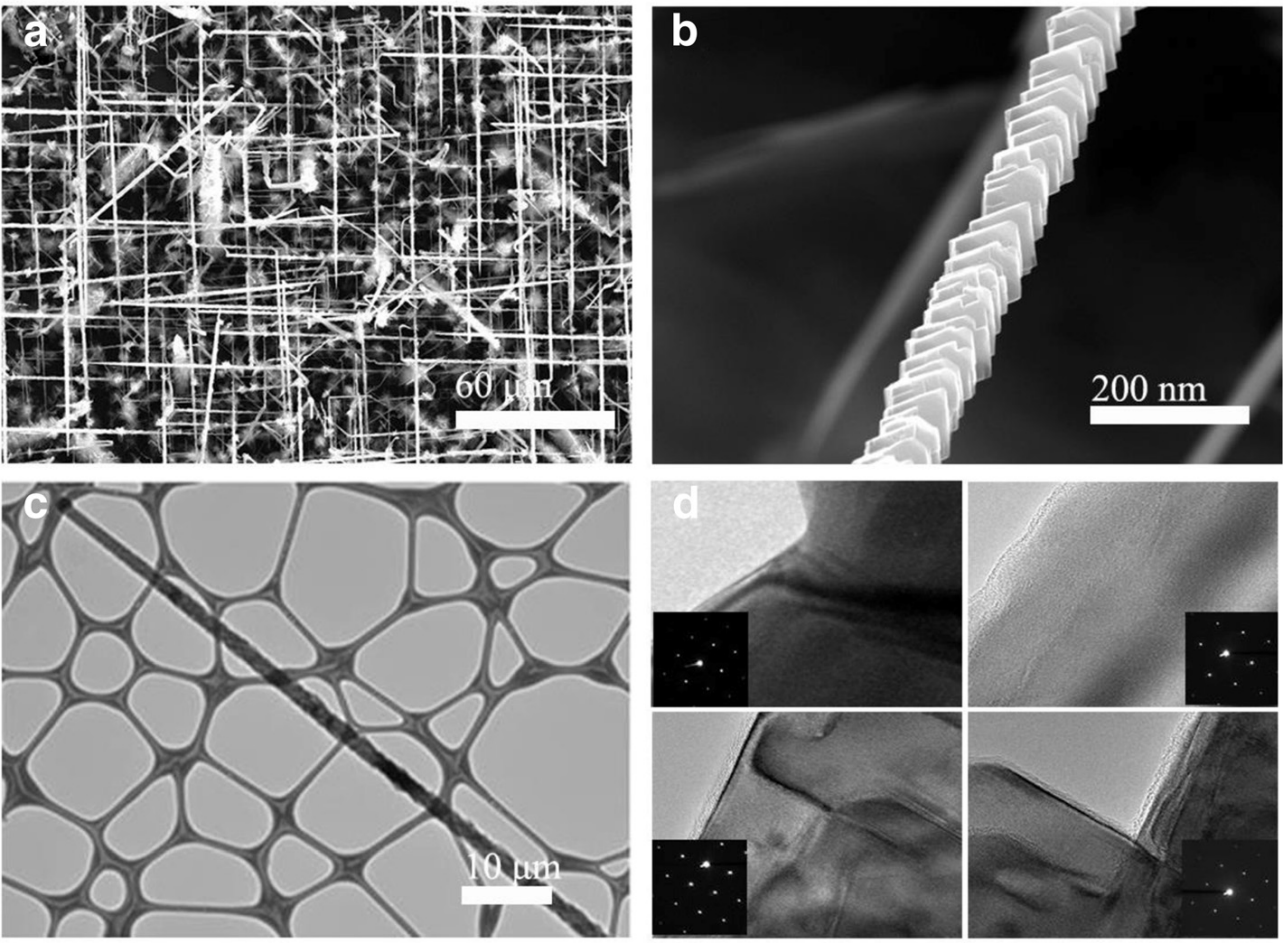
Rough SiNWs using Mn as catalyst. **(a)** A SEM image of rough-surfaced SiNWS grown on Si (100) substrate. **(b)** Low-magnification SEM image of rough-surfaced SiNW. **(c)** TEM image of rough SiNW with a typical diameter of 300 nm at the bottom to 60 nm at the tip. **(d)** HR-TEM images of rough-surfaced SiNW from top to bottom.

SiNWs were also grown on a Si (100) substrate using a combination of Mn and high gas flow rate. As reported previously [40,41], Figure 3a shows a SEM image of the SiNWs grown on the substrate with the assistance of Mn catalyst under 3,000 sccm∙min^−1^ of H_2_. The SiNWs grew in the <111 > direction, tenths of μm in length and tenths of nm in diameter, relatively larger than the other NWs in this study. The SiNWs show globular droplets at their tips that indicate the operation of the VLS mechanism. The composition of globules was 64% of Si and 36% of Mn, and any Mn component was not found in the nanowires [40]. It should be noted that the SiNWs grown with Mn showed a rough surface compared to the other catalysts, as shown in Figure 3b,c. It should also be noted that the SiNWs were tapered with larger diameters toward the bottom. This indicates a sidewall deposition by the vapor-solid (VS) mechanism, which involves the direct deposition of Si atoms from the vapor, resulting in a roughened surface. This side growth may be caused by the high flow rate of gas that enhances the atom adsorption rate on crystalline surfaces by increasing effective collisions and impinging atoms from the vapor to the nanowire surface, inducing VS growth in the radial direction [42,43]. Nanoscale-controlled curvature radius could be controlled by a well-defined amorphous SiO2 layer/Si [44]. The side wall roughening has been observed in the previous study by using Au-Si-Mn catalysts [45]; however, the roughening mechanism is different. That is, the shape of catalyst leads the faceting of nanowires in the previous study; however, collisions and impinging of atoms leads the faceting in this study.

It should be noted that the VS growth precedes only in the Mn catalyst case, and this may be due to a slow growth rate of the NWs with the Mn catalyst. Our study indicates that the growth rate of the SiNWs with Mn catalysts is slow compared to other catalysts, such as Au or Pt [46], which could provide enough time for a sidewall growth [40,41]. The larger diameter of the NWs may also contribute to the side growth, since the Gibbs-Thompson effect plays a critical role for thinner nanowires that prevent the sidewall deposition over the course of the growth [47]. Figure 3d shows the detailed crystal structures of the rough SiNWs from the top to the bottom regions. The features observed in the fast Fourier transform (FFT) image of the rough SiNW indicate that the lowest surface energy facets, the {111} planes with a surface energy of 1.23 J/m^2^, were exposed. This suggests that the roughening of the NW surface contributes to minimizing the surface energy and, in turn, decreasing the stability of the SiNWs.

**Figure 4:**
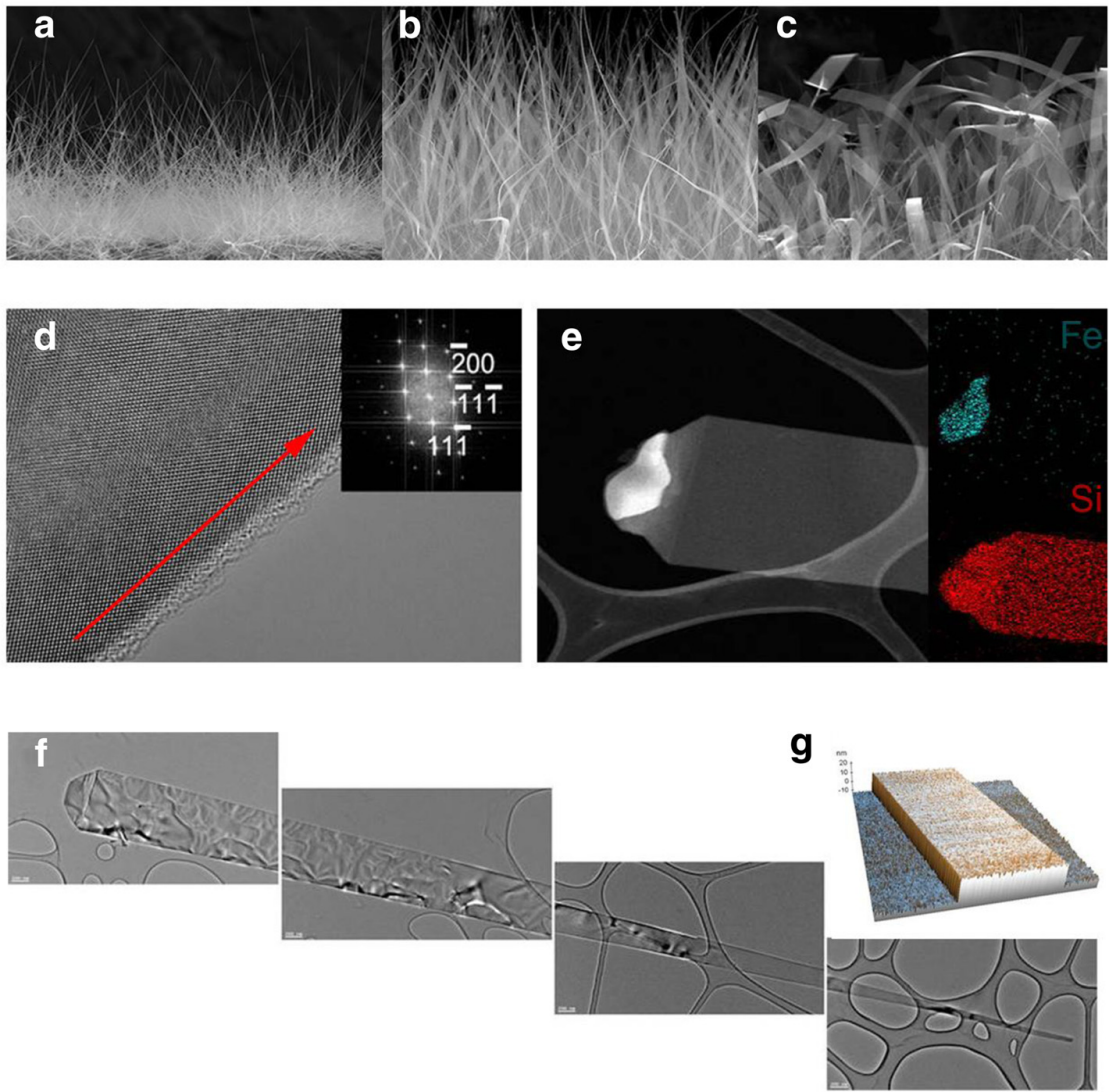
Ribbon-shaped SiNWs using Fe catalyst. **(a)** SEM image of ribbon-shaped SiNWs with 200 nm width at the bottom and at the top (H_2_ - 1,000 sccm∙min^−1^). **(b)** 5 μm width at the bottom and top (H_2_ - 2,000 sccm∙min^−1^). **(c)** 20 μm width at the bottom and top (H_2_ - 3,000 sccm∙min^−1^). **(d)** HR-TEM image of SiNW, showing single crystal with <100 > growth direction along [110] zone axis. **(e)** DES spectra of the SiNW and Fe catalyst. The data show that the body of the nanowire is Si and the metal alloy is Fe and Si component. **(f)** Low-magnification TEM image show that narrow bottom and wide top must lead the growth of the shape. **(g)** AFM image of SiNW. The thickness is about 20 nm.

Finally, ribbon-shaped SiNWs could be grown by using a Fe catalyst under a high gas flow rate. Figure 4a shows SiNWs grown under different flow rates. Under a low flow rate of H_2_, such as 1,000 sccm∙min^−1^, typical SiNWs were grown on the substrate (Figure 4a). Under a moderate H_2_ flow rate (2,000 sccm∙min^−1^), ribbon-shaped SiNWs with a width of about 3 μm were grown (Figure 4b). Under a high gas flow rate of 3,000 sccm∙min^−1^, these SiNWs have widths of 10 to 20 μm and lengths of hundreds of micrometers (Figure 4c). This clearly indicates that the width of the SiNWs increased with increasing flow rate. The SEM and TEM images (Figure 4d,e) also show that the VLS mechanism is operating for the growth of these ribbon-shaped SiNWs. A HRTEM analysis demonstrates that the SiNWs are single crystals with the <100 > growth direction. The inset of Figure 4e shows the elemental mapping of the iron and silicon X-ray intensity compositing along the nanowire. As shown in Figure 4f, the width of SiNW is thicker, with a typical width of 40 nm at the bottom (right) to 20 μm at the tip (left). The thicknesses of these ribbon-shaped SiNWs are approximately 16 to 20 nm (Figure 4g).

The growth of ribbon-shaped SiNWs by the VLS mechanism is interesting since the liquid catalyst globule generally results in rod-shaped SiNWs for the majority of instances. It should be noted that the shape of the catalyst could itself be ribbon-like, as shown in Figure 4c, thus leading to the growth of ribbon-shaped SiNWs.

In this study, the Fe component is supplied to the substrate through the vapor phase continuously with the SiCl_4_; thus, they dissolve and alloy together, forming liquid droplets during the course of the growth. In the conditions, the dissolving rate of Fe and Si would be different, which would produce a compositional gradient in the Fe-Si droplet. The compositional gradient may not be significant under a low gas flow rate; however, it will likely be significant under a high flow rate because the dissolution rate of the metal components directly depends on the vapor impinging on the liquid catalyst. This gradient will result in the precipitation of Si asymmetrically at the solid-liquid interface, i.e., the precipitation of Si mainly occurred at the Si-rich sides, resulting in the widening of the SiNWs to form ribbons.

## Conclusions

In this study, we demonstrated that various SiNWs could be grown by simply combining a high flow rate of gas with different types of catalyst. Considering the limitations of the VLS mechanism for a structural modulation of SiNWs, our combinatorial approach provides a new path for this modulation. It also provides a route to study the unusual VLS growth of SiNWs under high gas flow rates, i.e., highly dynamic conditions, that should be helpful toward advancing devices in several applications.

## Abbreviations

EDS: energy-dispersive spectroscopy
SEM: scanning electron microscopy
SiNWs: silicon nanowires
TEM: transmission emission microscopy
VLS: vapor-liquid-solid
